# Perioperative Anxiety and Stress Cortisol on Postoperative Pain: Pharmacological and Non-Pharmacological Management Strategies

**Published:** 2026-04-30

**Authors:** Aren Yarcan, Alexander Abdou, Devendra K Agrawal

**Affiliations:** 1 Alabama College of Osteopathic Medicine, Dothan, AL 36303, USA; 2 Department of Translational Research, College of Osteopathic Medicine of the Pacific,Western University of Health Sciences, Pomona, CA 91766, USA

**Keywords:** Postoperative Pain, Perioperative Anxiety, Psychological Stress, Cortisol, Hypothalamic-Pituitary-Adrenal Axis, Pain Perception, Perioperative Care, Multimodal Analgesia

## Abstract

Postoperative pain remains a significant clinical challenge associated with delayed recovery, prolonged rehabilitation, and increased healthcare utilization. Despite advances in analgesic techniques, outcomes remain variable, suggesting that non-physical factors such as perioperative stress and anxiety play an important role. A relationship between perioperative anxiety, stress, and postoperative pain was examined with emphasis on physiologic mechanisms, assessment strategies, and management approaches. A narrative review was conducted using PubMed searches of the studies published in last 25 years. Search terms included cortisol, stress, perioperative surgery, postoperative pain, sex differences, questionnaires, and therapeutic interventions. Peer-reviewed studies involving human subjects were included if they evaluated perioperative stress, cortisol dynamics, postoperative pain, or related interventions. Preoperative anxiety is consistently associated with increased postoperative pain and higher analgesic requirements. Activation of the Hypothalamic-Pituitary-Adrenal Axis and subsequent cortisol release represents a key mechanism linking stress to pain modulation; however, significant variability limits the role of cortisol as a standalone biomarker. Validated tools such as the Brief Measure of Emotional Preoperative Stress, State-Trait Anxiety Inventory, and Hospital Anxiety and Depression Scale can identify high-risk patients but remain underutilized. Sex differences are evident, with women reporting higher pain scores and stronger associations between anxiety and postoperative pain. Non-pharmacological interventions, including music therapy and perioperative communication, demonstrate modest benefit, while pharmacological strategies show variable efficacy. Perioperative stress and anxiety significantly influence postoperative pain outcomes. A multimodal, patient-centered approach incorporating structured assessment and targeted interventions may improve recovery and optimize perioperative care.

## Introduction

Pain is both a necessary physiological function and a significant pathological process, posing significant health risks [1-3]. Defects in the endogenous pain-inhibitory pathways are major factors in the initiation and chronicity of pain [4,5]. Multimodal approaches and psychological treatments have been used in the management of pain [6-11]. Indeed, electromagnetic field therapy as a non-pharmacological strategy has been effective in relieving pain in various conditions [12,13]. However, postoperative pain remains a significant clinical challenge that impacts surgical outcomes, often leading to delayed wound healing, prolonged rehabilitation, and reduced mobility [14]. Despite advances in analgesic techniques, postoperative pain remains a persistent clinical challenge, with evidence supporting the role of pharmacological strategies in reducing pain and opioid use [15]. Preoperative anxiety has also been associated with prolonged hospital stays [16], highlighting the potential influence of psychological factors in the perioperative period. While the physical mechanisms of pain have been extensively studied, the contribution of psychological and physiological stress to pain perception and recovery warrants further investigation. Perioperative anxiety can be understood as a transient psychological state characterized by excessive worry related to an upcoming surgical procedure. In this critical review, psychological stress refers to the cognitive appraisal of a perceived stressor, whereas anxiety reflects the emotional response to that stressor. Physiological stress refers to measurable biological responses, including changes in heart rate, blood pressure, and cortisol levels. These processes exist along a continuum, as psychological stress and anxiety may contribute to activation of physiological stress pathways. It should be noted that terminology across the literature is not uniform, and some studies use “stress” or “psychological stress” to describe emotional experiences like anxiety. Stress plays an important role in pain processing [17]. Activation of the hypothalamic-pituitary-adrenal (HPA) axis results in cortisol release, a key mediator of the physiological stress response [18]. Stress-related hormonal alterations may play an important role in shaping postoperative pain perception, with acute psychological stress associated with elevations in cortisol that may influence pain sensitivity and pain thresholds [19]. Recent studies have explored the use of salivary cortisol as a biomarker of stress in the perioperative setting [20]. While cortisol measurement may provide insight into physiological stress, variability in collection methods and patient factors limits its routine clinical application. Additionally, it is important to note that preoperative pain itself may influence both stress responses and postoperative pain outcomes, which may limit the utility of cortisol as a standalone predictor. This review aims to examine the relationship between perioperative anxiety, stress, and postoperative pain, and to evaluate both pharmacological and non-pharmacological management strategies. Additionally, we discuss commonly used tools for assessing perioperative anxiety and stress, as well as explore sex and gender differences in perioperative pain and stress responses. The goal is to provide a more comprehensive framework for optimizing perioperative care.

### Search Methods

We conducted PubMed searches in March (2026) for the studies published in the last 25 years using key terms including cortisol, stress, perioperative surgery, postoperative pain, sex differences, questionnaires, and both pharmacological and non-pharmacological interventions. We included peer-reviewed studies involving human subjects that focused on perioperative stress, cortisol levels, postoperative pain, and related interventions. Studies involving non-human subjects were excluded. We independently selected articles for inclusion. Relevant data extracted included study design, sample size, participant demographics, interventions, and key findings related to perioperative stress and postoperative outcomes.

### Correlation Between Stress, Cortisol, and Postoperative Pain

The relationship between perioperative anxiety, cortisol secretion, and postoperative pain represents an important factor influencing surgical outcomes. Existing literature suggests that preoperative anxiety may influence postoperative pain perception and recovery. For example, a study involving adolescents undergoing scoliosis surgery identified a correlation between preoperative psychological stress, measured using questionnaire-based assessments, and postoperative pain intensity on the third postoperative day [21]. These findings highlight the importance of incorporating psychological stress assessments into preoperative evaluation. Cortisol, a hormone released in response to physiological stress, plays a pivotal role in mediating this relationship. Elevated cortisol levels have been correlated with increased postoperative pain, suggesting that cortisol may act as a key mediator in the stress pain pathway [19,22]. In an experimental model, Choi et al. [19] induced psychological stress by having participants perform a simulated medical test, while pain was elicited using electrical stimulation. Under these conditions, stress was associated with increased anxiety ratings, elevated cortisol levels, and increased pain sensitivity, reflected by higher pain ratings and reduced pain thresholds. In contrast, Kanegane et al. [22] did not find an association between cortisol and anxiety but did observe higher cortisol levels in patients reporting pain. The mechanism by which cortisol enhances pain sensitivity may involve its effects on immune function, inflammation, and central nervous system processing [23]. A narrative review by Manou-Stathopoulou et al. questions the traditional model of the perioperative stress response and suggests it may not fully reflect what is seen in clinical practice [24]. The authors note that cortisol levels can vary widely between patients and are influenced by baseline health, chronic stress, and existing comorbidities even before surgery. They also highlight that postoperative factors such as immobility, nutrition, and complications like infection can further alter cortisol levels. In addition, the way cortisol is measured may not reliably reflect true hypothalamic-pituitary-adrenal axis activity due to its dynamic nature and variability between assays. It is also worth noting that as many as 50% of individuals with major depression exhibit increased cortisol secretion, which may influence perioperative cortisol measurements [25,26]. Taken together, these factors suggest that cortisol levels alone may not provide a complete picture of the stress response and should be interpreted in the broader clinical context.

### Sex/Gender Differences in Perioperative Pain and Stress

Sex and gender differences appear to play an important role in perioperative pain and stress responses. It has been reported that women experience more severe and longer lasting postoperative pain than men, with higher pain scores observed at multiple postoperative timepoints [27,28]. These differences may reflect both biological and psychosocial contributors to pain perception and recovery. Women also appear to report greater psychological distress in response to surgery, although physiologic stress responses may differ by measure. For example, some studies suggest that women experience higher perceived stress, whereas men demonstrate greater cortisol increases under stress conditions [29]. This distinction further supports the importance of separating subjective psychological stress from physiologic stress responses when interpreting perioperative findings in the clinical setting. The relationship between preoperative anxiety and postoperative pain has also been reported to be stronger in women than in men, particularly in the development of both acute and chronic postsurgical pain [30]. This suggests that anxiety may have a greater effect on pain outcomes in female patients and may be especially relevant when identifying patients at risk for prolonged recovery or persistent pain. Differences in analgesic requirements have also been described. Some studies report that women require approximately 30% more morphine than men to achieve similar analgesia [31], although other studies using enhanced recovery protocols have found no significant difference in opioid consumption despite higher reported pain scores in women [32]. Together, these findings suggest that pain intensity and analgesic use may not always align and should be interpreted within the broader perioperative context. Important confounders must also be considered when interpreting sex differences. Age and preoperative chronic pain appear to substantially influence these relationships, with differences being most pronounced in women over 50 years of age and in patients with preoperative pain [33]. These factors may partly explain why findings are not uniform across studies. Emerging mechanistic work provides a possible biologic explanation for these differences. Prolactin mediated nociceptor sensitization has been proposed as one pathway that may contribute to sex differences in stress and pain signaling [34]. This may help explain why women appear to be more susceptible to certain stress related pain responses, though further research is needed to clarify its clinical relevance. Overall, these findings highlight the importance of incorporating sex and gender specific considerations into perioperative assessment. Recognizing how psychological stress, physiologic responses, and pain perception differ between patients may improve the identification of individuals at higher risk for adverse pain outcomes. Given these findings, incorporating structured assessment of perioperative anxiety and stress into preoperative evaluation may help identify patients at risk for increased postoperative pain and inform the use of validated assessment tools and targeted interventions.

### Approaches to Assess Perioperative Psychological Stress

The psychological impact of major surgical procedures on patients is multifaceted, which necessitates a deeper investigation into the psychological factors that influence perioperative outcomes. Stress assessment tools are essential for identifying patients at risk of heightened stress, which can lead to increased postoperative pain. Among these tools, the Brief Measure of Emotional Preoperative Stress (B-MEPS) was specifically developed for preoperative psychological assessment. The B-MEPS was designed to evaluate individuals particularly vulnerable to emotional stress in the perioperative context. This tool has undergone further revisions and rigorous testing to assess its accuracy and reliability. A recent study demonstrated that the refined B-MEPS is not only a valid measure of preoperative stress but also a predictive tool for postoperative outcomes [35]. The study revealed that patients with high scores on the refined B-MEPS were more likely to experience moderate to severe pain 24 hours post-surgery. Additionally, these patients required higher doses of morphine 12 hours postoperatively compared to those with lower B-MEPS scores. These findings underscore the importance of integrating psychological assessments into preoperative care. The refined B-MEPS offers a practical, standardized method for identifying high-risk patients, allowing clinicians to implement targeted interventions to reduce stress, potentially mitigating postoperative pain and enhancing recovery. The integration of technology into stress assessment presents a promising frontier for enhancing the precision and efficiency of preoperative evaluations. Digital platforms and smartphone applications can streamline the administration of tools like the refined B-MEPS, enabling real-time monitoring of patients’ stress levels. For instance, Yang et al. [36] employed a smartphone app utilizing Ecological Momentary Assessment (EMA) to track mood and stress fluctuations in Moyamoya disease patients over a week, identifying key factors influencing their psychological state. Digital solutions empower patients to actively participate in their preoperative care through self-assessment, potentially leading to improvement in surgical outcomes and patient satisfaction scores. Future research should focus on validating these digital assessment methods and exploring their integration with existing healthcare systems to maximize their benefits in perioperative care. Preoperative anxiety, which affects up to 80% of patients undergoing high-risk surgeries, has been linked to adverse psychological and somatic outcomes, influencing anesthesia management, postoperative care, and overall recovery [37]. In a multicenter cross-sectional study, preoperative anxiety was identified in 15.8% of Chinese adult patients undergoing elective surgeries, as measured using the 7-item Perioperative Anxiety Scale (PAS-7) [38]. Factors associated with higher anxiety in this study included female sex, younger age, first-time surgery, higher surgical risk, and poor preoperative sleep, highlighting the role of both patient characteristics and perioperative context in shaping their level of anxiety. Reliable psychometric scales are essential for assessing anxiety levels in the perioperative setting. Some questionnaires being employed in both the clinical and research settings to assess anxiety include the State-Trait Anxiety Inventory (STAI), the Hospital Anxiety and Depression Scale (HADS), and the Amsterdam Preoperative Anxiety and Information Scale (APAIS), in combination with the visual analogue scale (VAS) and Patient-Reported Outcomes Measurement Information System (PROMIS) Pain Interference (PROMIS-PI) scores for pain assessments. The State-Trait Anxiety Inventory (STAI) has been extensively used to assess preoperative anxiety. One study utilizing STAI scores explored the relationship between caregiver and patient anxiety and found positive associations in male caregiver–patient pairs; however, these findings were not statistically significant, suggesting only a potential influence rather than a confirmed relationship [39]. These findings suggest that psychosocial interactions may play a role in perioperative anxiety, but their impact is inconsistent and context dependent. This highlights the utility of tools such as the STAI in identifying potential psychosocial factors that may influence perioperative anxiety and postoperative outcomes, even when relationships are not consistently demonstrated across studies. Another study using STAI and VAS found that preoperative state anxiety was not associated with overall or resting pain scores at six weeks following surgery for pectus excavatum. However, higher preoperative anxiety was associated with increased pain during activity at the same follow-up period of 6 weeks [40]. The HADS has been used to assess anxiety before and after arthroplasty [41] and to evaluate anxiety and depression in postoperative gastric cancer patients undergoing reminiscence therapy, a non-pharmacological intervention aimed at improving emotional well-being [42]. These findings highlight the role of HADS not only in measuring perioperative psychological states but also in evaluating the impact of targeted interventions on patient outcomes. The VAS has been utilized to assess pain levels and identify key risk factors for acute pain in elderly patients following laparoscopic radical resection of colorectal cancer [43] further supporting the use of standardized tools in understanding both psychological and pain-related outcomes. Patient-Reported Outcomes Measurement Information System (PROMIS) Pain Interference (PROMIS-PI) scores have also proven crucial in assessing surgical outcomes. In a study of patients who underwent surgery for de Quervain’s tenosynovitis, higher PROMIS Pain Interference scores were associated with worse pain and functional outcomes, highlighting the importance of considering these scores and psychosocial factors before reintervention [44]. The Monitoring Subscale of the Miller Behavioral Style Scale (C-MMBSS) has been effectively used in preoperative education to reduce anxiety and depression and to improve patient satisfaction with the education provided [45]. Furthermore, the C-MMBSS, a Chinese translated version, showed good content and construct validity after being refined to a 26-item scale, with the Monitoring subscale demonstrating acceptable internal consistency in patients undergoing percutaneous coronary intervention and their caregivers [45,46]. Its adaptation to keep into account cultural differences in understanding and learning styles adds to its strength as a tool for assessing coping styles and helping guide more tailored patient communication. Despite the demonstrated utility of these assessment tools, their use in clinical practice remains variable. A survey of U.S. spine surgeons revealed that only 37% use presurgical psychological screening (PPS), with more experienced surgeons and those with higher case volumes being more likely to implement PPS, despite strong beliefs in its impact on outcomes such as pain relief and therapy adherence [47]. In conclusion, a range of questionnaires and scales are available to identify and assess perioperative stress in patients. These tools help in understanding patient anxiety, predicting surgical outcomes, and customizing interventions to improve care (table 1), presents a detailed overview of these questionnaires, highlighting their advantages and disadvantages.

### Non-pharmacological Therapy to Manage Stress

To optimize perioperative care, non-pharmacological interventions should be considered as adjunctive therapies within a holistic approach to pain and stress management. While pharmacological treatments remain central to postoperative pain control, integrating complementary therapies may enhance patient outcomes by addressing the psychological and emotional dimensions of pain. A systematic review by Tola et al. [61] found that interventions such as acupuncture, music therapy, and aromatherapy can reduce preoperative anxiety and postoperative pain. In women undergoing breast cancer surgery, music demonstrated the most consistent and larger effect sizes, while aromatherapy showed more modest effects and acupuncture demonstrated moderate benefit for postoperative pain. However, the limited number of studies available for each intervention restricts direct comparison and highlights the need for further research. Music therapy has been shown to alleviate anxiety before surgery and contribute to lower postoperative pain scores [62] with additional evidence suggesting that intraoperative music delivered via headset may reduce analgesic use and perioperative pain [63]. Similarly, aromatherapy, which involves the use of essential oils such as lavender for their calming properties, can be administered through inhalation or massage to promote relaxation. Tola et al. [61] found that aromatherapy was associated with modest reductions in preoperative stress, whereas massage demonstrated inconsistent or limited effects across the included studies.

In pediatric patients, where stress and anxiety are often more difficult to assess and manage, non-pharmacological interventions are also important. Perioperative dialogue (PD) techniques have been shown to lower salivary cortisol levels and reduce morphine requirements in children undergoing surgery [64]. This approach involves consistent communication with the same caregiver throughout the perioperative period, helping prepare the child for upcoming events and address concerns. The effectiveness of these interventions supports the use of a more comprehensive approach to perioperative care. By addressing psychological stress alongside physical pain, non-pharmacological therapies may contribute to improved patient comfort and reduced reliance on opioids. As perioperative care continues to evolve, these strategies may serve as useful adjuncts in managing stress and optimizing recovery.

### Pharmacological Strategies- Preemptive Analgesia

Preemptive analgesia involves the administration of analgesics prior to surgical intervention to reduce the development of central sensitization; a mechanism associated with increased postoperative pain. By targeting pain pathways early, this approach has been associated with reductions in postoperative pain intensity, decreased opioid consumption, and a lower incidence of postoperative nausea and vomiting (PONV), a common side effect of opioid use [15]. Pharmacological strategies aimed at reducing perioperative anxiety and stress are an important component of preemptive care, as psychological distress may amplify pain perception and negatively impact recovery. A growing body of literature supports the use of multimodal approaches that combine anxiolysis with analgesia to improve perioperative outcomes [65,66]. Benzodiazepines remain among the most used preoperative anxiolytics. Midazolam is widely utilized due to its rapid onset and ability to produce anxiolysis, amnesia, and sedation through enhancement of GABA-A receptor activity [67]. However, unwanted reactions may occur, including paradoxical reactions, oversedation, and cognitive or psychomotor impairment, particularly in vulnerable populations [67,68]. Melatonin has emerged as a potential alternative for preoperative anxiolysis. Systematic review data suggest that melatonin is associated with reduced preoperative anxiety compared to placebo. It may provide similar anxiolytic effects to benzodiazepines with fewer cognitive side effects [68]. However, findings are not uniform across populations. The MAGIC trial and subsequent analyses demonstrated that melatonin was inferior to midazolam for anxiolysis in pediatric patients undergoing elective surgery. This highlighted important age-related differences in efficacy [69-70]. Alpha-2 adrenergic agonists, including dexmedetomidine and clonidine, offer another pharmacologic option for perioperative anxiolysis. These agents reduce sympathetic outflow, producing sedation and anxiolysis with minimal respiratory depression [67]. Dexmedetomidine has been used off-label for pediatric premedication via intranasal or oral routes, though its use may be limited by availability and hemodynamic effects.

Gabapentinoids, including gabapentin and pregabalin, have also been studied for their use in perioperative anxiety and pain modulation. Meta-analytic data suggest moderate efficacy in reducing preoperative anxiety compared to placebo [71]. Pregabalin, particularly at doses around 150 mg, has been associated with reduced anxiety, improved sleep quality, and decreased postoperative pain in certain surgical populations [72,73]. Similarly, gabapentin has been shown to reduce preoperative anxiety and pain catastrophizing in highly anxious patients; however, this benefit often comes with increased sedation [74]. Despite these findings, the use of gabapentinoids remains controversial due to concerns regarding respiratory depression, particularly when combined with opioids. Furthermore, conflicting clinical trial data limit their widespread adoption [75] Some studies have also failed to demonstrate that pregabalin is not inferior compared to traditional anxiolytics such as diazepam [76]. Antidepressants, including selective serotonin reuptake inhibitors (SSRIs) and serotonin-norepinephrine reuptake inhibitors (SNRIs), are effective for the treatment of anxiety disorders. Despite this, their delayed onset of action, often requiring several weeks, limits their use in acute perioperative settings. Overall, pharmacological strategies for perioperative anxiety and pain management should be individualized based on patient characteristics, surgical context, and risk factors. Multiple agents to achieve this are effective; however, variations in outcomes and potential adverse effects highlight the importance of a multimodal and patient-centered approach.

## Discussion

This review highlights the complex and multifactorial relationship between perioperative stress, anxiety, and postoperative pain. Findings across physiologic mechanisms, assessment strategies, and both pharmacological and non-pharmacological interventions are discussed. While cortisol has traditionally been viewed as a central mediator of the stress response, the evidence suggests that its role in postoperative pain is not uniform or easily interpreted. Variability in cortisol dynamics, patient comorbidities, and perioperative factors limits its reliability as a standalone biomarker. A more complete picture including the interaction between psychological stress, physiologic response, and patient-specific factors appears to be more clinically relevant than any single measurement. A consistent finding across the literature is the importance of psychological factors, particularly preoperative anxiety, in shaping postoperative pain outcomes. This relationship is further influenced by sex-specific differences, with women demonstrating higher pain scores, greater perceived stress, and a stronger association between anxiety and postoperative pain. These findings suggest that perioperative stress and pain should not be approached as uniform processes, but rather as individualized responses influenced by both biologic and psychosocial variables. The use of structured assessment tools represents a practical approach to identifying patients at risk. Instruments such as the B-MEPS, STAI, and HADS can capture perioperative anxiety and stress, and in some cases, predict postoperative pain and analgesic requirements. Despite their efficacy, these tools remain underutilized in clinical practice. This gap between evidence and implementation highlights an opportunity to incorporate standardized screening into routine preoperative workflows. This can be particularly useful for patients at higher risk, including those with high baseline anxiety, chronic pain, or undergoing high-risk surgical procedures. From a clinical perspective, these findings support a more targeted approach to perioperative care. Patients with elevated anxiety scores or known risk factors may benefit from early intervention strategies. Non-pharmacological approaches, such as music therapy, aromatherapy, and perioperative dialogue, offer low-risk adjuncts that may reduce anxiety and improve pain outcomes, particularly in vulnerable populations such as pediatric patients. Pharmacological strategies, including benzodiazepines, melatonin, alpha-2 agonists, and gabapentinoids, provide additional options, though their use must be individualized based on patient characteristics and risk profiles. Importantly, the variability in efficacy and side effect profiles across these agents underscores the need for a multimodal approach rather than reliance on a single intervention. Despite these advances, several gaps in the literature remain. There is a lack of standardized protocols for measuring cortisol in the perioperative setting, limiting comparability across studies and clinical applicability. Additionally, few studies directly compare pharmacological and non-pharmacological interventions in head-to-head trials, making it difficult to determine optimal treatment strategies. Many studies also focus on specific populations or procedures, reducing generalizability. Furthermore, gender-specific analyses are often secondary or underpowered, despite clear evidence that gender influences both stress and pain responses. Future research should focus on developing standardized frameworks for assessing perioperative stress that integrate both psychological and physiologic measures. Prospective studies evaluating combined intervention strategies, including both pharmacological and non-pharmacological approaches, are needed to better define optimal care pathways. Greater emphasis should also be placed on sex-specific mechanisms and outcomes, as well as on identifying biomarkers that more reliably reflect the stress-pain relationship beyond cortisol alone. In conclusion, perioperative stress and anxiety play a significant role in postoperative pain and recovery, but their effects are variable and influenced by multiple patient-specific factors. A structured, individualized approach that incorporates validated assessment tools and multimodal intervention strategies may improve patient outcomes. Advancing the field will require more standardized methodologies, comparative effectiveness research, and a deeper understanding of the biologic and psychosocial mechanisms underlying the stress-pain relationship.

### Limitations

This review has several limitations. The included studies demonstrate heterogeneity in design, patient populations, and outcome measures, which limits direct comparison and synthesis. Many findings are derived from observational studies or secondary analyses, reducing the strength of causal inference. Additionally, variability in how stress and anxiety are defined and measured across studies introduces further complexity. The narrative nature of this review, while allowing for broad synthesis, may also introduce selection bias and limit the ability to perform quantitative comparisons.

### Future Directions and Implications

Future research in perioperative care should address several critical areas. The development of precise and rapid stress assessment tools for both elective and emergent surgical settings warrant further investigation. Longitudinal studies are needed to elucidate the long-term effects of perioperative stress management on postoperative outcomes, including pain intensity, opioid consumption, length of hospitalization, and patient satisfaction. Additionally, exploration of sex-based differences in stress and pain responses may inform more personalized approaches to perioperative care. Further examination of the feasibility and clinical utility of routine cortisol measurement in surgical patients is warranted. The integration of technological innovations, such as smartphone applications for stress assessment and monitoring, may enhance perioperative care and should be evaluated. Lastly, large-scale, multicenter randomized controlled trials are necessary to validate the efficacy of combined pharmacological and non-pharmacological interventions in managing perioperative stress and pain. These studies could potentially lead to the development of standardized protocols aimed at improving overall surgical outcomes.

## Figures and Tables

**Figure 1:**
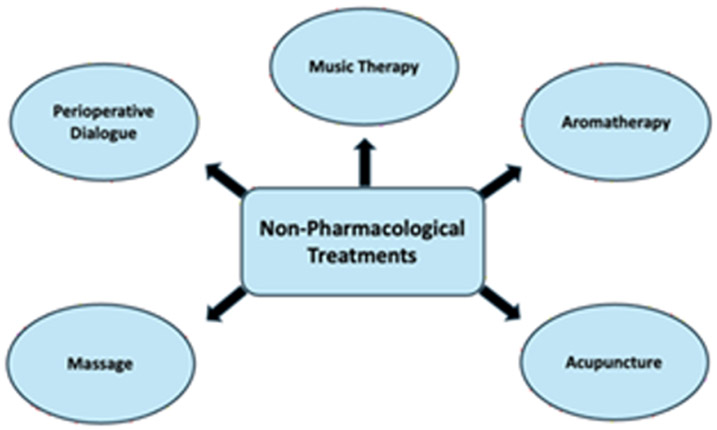
Non-Pharmacological Treatments: Overview of nonpharmacological strategies used to address perioperative stress and pain, which target the psychological and emotional components of the stress response to improve postoperative outcomes.

**Figure 2:**
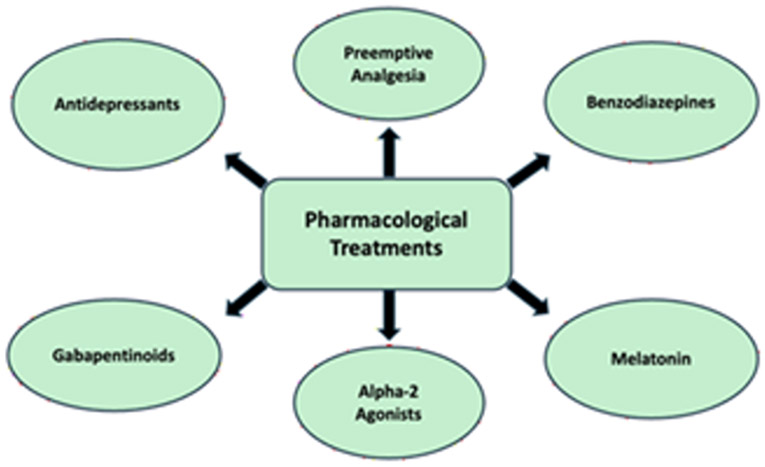
Pharmacological Treatments: Overview of pharmacological strategies used to address perioperative stress and pain management, which aim to modulate pain pathways and reduce anxiety in the perioperative period.

**Table 1:** An overview of the advantages and disadvantages of commonly used preoperative psychological assessment tools and questionnaires for predicting postoperative pain and stress outcomes as reported in several studies [45-60].

Questionnaire	Advantages	Disadvantages
Brief Measure of Emotional Preoperative Stress (B-MEPS)	· Provides a broad emotional evaluation, as opposed to solely screening anxiety/ depression [48]	· Preoperative emotional stress is only one of several factors that can contribute to postoperative pain [49]
	· Brief with 12 items and suitable to predict individuals prone to severe postoperative pain [48]	· To establish cross-cultural validity and reliability, additional studies using other language versions are essential [49]
State-Trait Anxiety Inventory (STAI)	· Differentiates between state (temporary) anxiety and trait (long-standing) anxiety [50]	· Relatively long at 40 items, length may inflate scores due to test anxiety [51]
	· Widely used and available in over 30 languages [50]	· Does not differentiate between anxiety and depression [51]
Hospital Anxiety and Depression Scale (HADS)	· Very brief, easy to use screening measure at 14 items [52]	· Reduced validity in some populations, particularly in the elderly [52]
	· Assesses both anxiety and depression [52]	· May not capture disease-specific anxieties [52]
Amsterdam Preoperative Anxiety and Information Scale (APAIS)	· Short and quick self-completion tool with only 6 items [53]	· Limited to preoperative situation, does not assess trait anxiety [55]
	· Successfully translated and validated in multiple languages and cultures [54]	· Does not distinguish well between fear of anesthesia and fear of surgery [56]
Visual Analogue Scale (VAS).	-Obtains measurements with more variability on a line continuum [57]	-Sensitive to format changes, horizontal scales more sensitive than vertical [58]
	-Simple format minimizes cognitive burden on patients [58]	-Wide variability in test-retest reliability [58]
Monitoring Subscale of the Miller Behavioral Style Scale (MMBSS)	· Useful for predicting informational search under threat [59]	· Time consuming with full survey at 32 items [45]
	· Men and women showed similar patterns in coping-style scores, with no statistically significant differences between the genders [59]	· Limited evidence on whether MBBS scores accurately predict real-world behavior during actual threat situations [60]
Presurgical psychological screening (PPS)	· Improved patient selection for better outcomes in spine surgery [47]	· Variability in methods as there is no standardized approach to PPS [47]
	· Facilitates a customized treatment plan tailored to individual patient needs [47]	· Difficult to implement since PPS is outsourced to psychologist [47]

**Note:** All survey metrics for anxiety suffer due to self-report bias except for presurgical psychological screening since this evaluation is completed by a psychologist.

## Key Points

1. Postoperative pain remains a persistent clinical challenge, contributing to delayed recovery and increased healthcare utilization.
2. Preoperative anxiety is common and consistently associated with increased postoperative pain and higher analgesic requirements.
3. Psychological and physiological stress exist on a continuum and jointly influence pain perception and recovery.
4. Activation of the Hypothalamic-Pituitary-Adrenal Axis links stress to pain modulation through cortisol release.
5. Cortisol demonstrates high interindividual variability and is influenced by comorbidities and perioperative factors, limiting its role as a reliable standalone biomarker.
6. Validated tools (e.g., B-MEPS, STAI, HADS) can identify high-risk patients but are underutilized in routine clinical practice.
7. Sex differences are significant, with women showing higher pain scores and stronger associations between anxiety and postoperative pain.
8. Non-pharmacological interventions (e.g., music therapy, perioperative communication) provide effective, low-risk adjuncts for reducing anxiety and pain.
9. Pharmacological strategies (e.g., benzodiazepines, melatonin, alpha-2 agonists, gabapentinoids) show variable efficacy and require individualized use.
10. A multimodal, patient-centered approach integrating psychological assessment and targeted interventions is essential to optimize perioperative outcomes.
